# Prevalence and risk factors for long COVID among cancer patients: a systematic review and meta-analysis

**DOI:** 10.3389/fonc.2024.1506366

**Published:** 2025-01-15

**Authors:** Hongkun Xu, Tingting Lu, Yajie Liu, Jingqi Yang, Simeng Ren, Baojin Han, Honghao Lai, Long Ge, Jie Liu

**Affiliations:** ^1^ Graduate School, Beijing University of Chinese Medicine, Beijing, China; ^2^ Guang’anmen Hospital, China Academy of Chinese Medical Sciences, Beijing, China; ^3^ Institute of Basic Research in Clinical Medicine, China Academy of Chinese Medical Sciences, Beijing, China; ^4^ Evidence-Based Social Science Research Center, School of Public Health, Lanzhou University, Lanzhou, China

**Keywords:** cancer, COVID-19, long COVID, prevalence, risk factors

## Abstract

**Objective:**

The prevalence of long COVID among cancer patients remains unknown. This study aimed to determine the prevalence of long COVID and explore potential risk factors among cancer patients.

**Methods:**

A systematic search was performed on PubMed, Web of Science, and Embase from database inception until 21 March 2024, to identify studies that reported long COVID in cancer patients. Two investigators independently screened the studies and extracted all information about long COVID in cancer patients for subsequent analysis. Methodological quality was assessed using the “Joannagen Briggs Institute (JBI) Critical Appraisal Checklist for Studies Reporting Prevalence Data”.

**Results:**

A total of 13 studies involving 6,653 patients were included. The pooled prevalence of long COVID was 23.52% [95% confidence interval (CI), 12.14% to 40.64%] among cancer patients reported experiencing long COVID after acute severe acute respiratory syndrome coronavirus 2 (SARS-CoV-2) infection. The pooled prevalence of any long COVID in cancer patients was 20.51% (95% CI, 15.91% to 26.03%), 15.79% (95% CI, 11.39% to 21.47%), and 12.54% (95% CI, 6.38% to 23.18%) in 3, 6, and 12 months follow-up duration. Fatigue was the most common symptom, followed by respiratory symptoms, myalgia, and sleep disturbance. Patients with comorbidities had a significantly higher risk of experiencing long COVID [odds ratio (OR) = 1.72; 95% CI, 1.09 to 2.70; *p* = 0.019]. No statistically significant differences in sex, primary tumor, or tumor stage were detected.

**Conclusion:**

Nearly a quarter of cancer patients will experience long COVID after surviving from SARS-CoV-2 infection, and this would even last for 1 year or longer. Fatigue, respiratory symptoms, myalgia, and sleep disturbance need to be more addressed and managed to reduce symptom burden on cancer patients and improve quality of life. Patients with comorbidities are at a high risk of developing long COVID. Further randomized controlled trials with rigorous methodological designs and large sample sizes are needed for future validation.

**Systematic review registration:**

https://www.crd.york.ac.uk/PROSPERO/, identifier CRD42023456665.

## 1 Introduction

Severe acute respiratory syndrome coronavirus 2 (SARS-CoV-2) continues to spread rapidly worldwide. According to the World Health Organization (WHO) Coronavirus (COVID-19) Dashboard, there have been over 772 million confirmed cases of COVID-19 worldwide, and nearly 7 million people have died from it (1). After recovery from acute infection, many patients will still experience persistent symptoms such as cough, dyspnea, and fatigue (2–4), which is known as long COVID. The prevalence of long COVID-19 is high (5, 6), independent of the severity of initial COVID-19 presentation (7). However, the definition of long COVID has not been universally agreed upon. “Long COVID” (includes both ongoing symptomatic COVID-19 from 4 to 12 weeks) was named by the UK National Institute for Health and Care Excellence (NICE) (8), “post-COVID-19 condition” (3 months from the onset of COVID-19 and with symptoms that last for at least 2 months and cannot be explained by an alternative diagnosis) was named by the WHO (9), and “Long COVID” (at least 4 weeks after infection is the start of when long COVID can first be identified) was named by the U.S. Centers for Disease Control and Prevention (CDC) (10), and approximately 65% of the studies do not use any of the three definitions (11).

Although 10%–30% of cancer patients die from SARS-CoV-2 infection (12–15), the majority of cancer patients recover from acute infection and are at risk of long COVID. One study revealed that compared with individuals not infected with COVID-19, patients with long COVID had greater mortality and healthcare utilization at the 1-year follow-up (16). Another study also revealed an association between long COVID and poorer survival outcomes in cancer patients (17), which may be related to providing less treatment, delaying treatment initiation, or promoting cancer progression (18). Understanding the long-term effects of COVID-19 in cancer patients is fundamental to trying to protect cancer patients from long COVID and the adverse events it may cause. However, because of the limited sample size and the wide variation in the reported prevalence of long COVID in cancer patients, no systematic reviews or meta-analyses have been published to address this issue.

Therefore, we conducted systematic reviews and meta-analyses of the existing evidence on long COVID, to determine the prevalence of long COVID and explore potential risk factors. The results of this study will help fill a significant knowledge gap about the lingering effects of COVID-19 on cancer patient outcomes.

## 2 Methods

This systematic review was performed according to the Preferred Reporting Items for Systematic Review and Meta-Analysis (PRISMA) (19), and the protocol was registered at PROSPERO (No. CRD42023456665).

### 2.1 Search strategy

Studies that assessed the long COVID among cancer patients were systematically searched in PubMed, Embase, and Web of Science from database inception to 21 March 2024. We used the following search terms: “COVID-19”, “Long COVID”, “Symptom”, and “Cancer”. The detailed search strategy is provided in **Supplementary Table S1**. The reference lists of the included studies were manually searched for additional studies that met the inclusion criteria.

### 2.2 Selection criteria

The inclusion criteria were as follows (1): include cancer patients infected with SARS-CoV-2; (2) measure long COVID symptoms; (3) assess symptoms at least 4 weeks after initial COVID-19 infection; and (4) provide data consisting entirely of cancer patients.

The exclusion criteria were as follows: (1) the study was a review, case report, conference abstract, or protocol; (2) the publications were repeated; (3) data or full texts were unavailable; and (4) non-English language publications.

### 2.3 Study selection and data extraction

Two investigators independently screened the titles and abstracts and removed irrelevant studies according to the inclusion and exclusion criteria. Two investigators extracted the following data: the characteristics of cancer patients, the test used for SARS-CoV-2 infection diagnosis, follow-up time, sample size, and outcomes. Any inconsistencies were resolved by discussion with a third investigator.

### 2.4 Quality assessment

Two investigators independently assessed methodological quality using the “Joannagen Briggs Institute (JBI) Critical Appraisal Checklist for Studies Reporting Prevalence Data” (20). The checklist consists of nine questions (1): Was the sample frame appropriate to address the target population? (2) Were study participants recruited in an appropriate way? (3) Was the sample size adequate? (4) Were the study subjects and setting described in detail? (5) Was data analysis conducted with sufficient coverage of the identified sample? (6) Were valid methods used for the identification of the condition? (7) Was the condition measured in a standard, reliable way for all participants? (8) Was there appropriate statistical analysis? and (9) Was the response rate adequate, and if not, was the low response rate managed appropriately? Each item has four options: yes, no, unclear, or not applicable. An overall score was used to reflect the number of questions with an option of “yes”.

### 2.5 Statistical analysis

We only used the study with the largest sample size for each meta-analysis if certain patients could be included in several studies. The long COVID ratio was used as the original proportion data. The Shapiro−Wilk normality test was employed to determine whether the data fit a normal distribution. If the measurement data conformed to a normal distribution, the original proportion was adopted for analysis; otherwise, the Freeman–Tukey double arcsine transformation was used to adjust the data. τ^2^ was used to estimate the between-study variance. The *I* ^2^ value was used to assess heterogeneity (21). If *I* ^2^ ≥ 50%, a random-effects model was used; otherwise, a fixed-effects model was used (22). The odds ratio (OR) and 95% confidence interval (CI) were used to pool dichotomous variables. Publication bias was examined using Egger’s test, and subgroup analyses were performed based on follow-up duration. Meta-analyses were pooled using the R Software (version 4.3.2 R Project for Statistical Computing, Vienna, Austria) and the “meta” R package (23). *p-*value < 0.05 was considered statistically significant.

For the analysis of each symptom, we found that most of the studies reported only the symptoms with a higher prevalence instead of all data on symptoms they investigate. Because it is inappropriate to combine these data directly, which might bias results, we used a systematic review to summarize symptoms with higher frequency.

## 3 Results

### 3.1 Literature search

**Figure 1:**
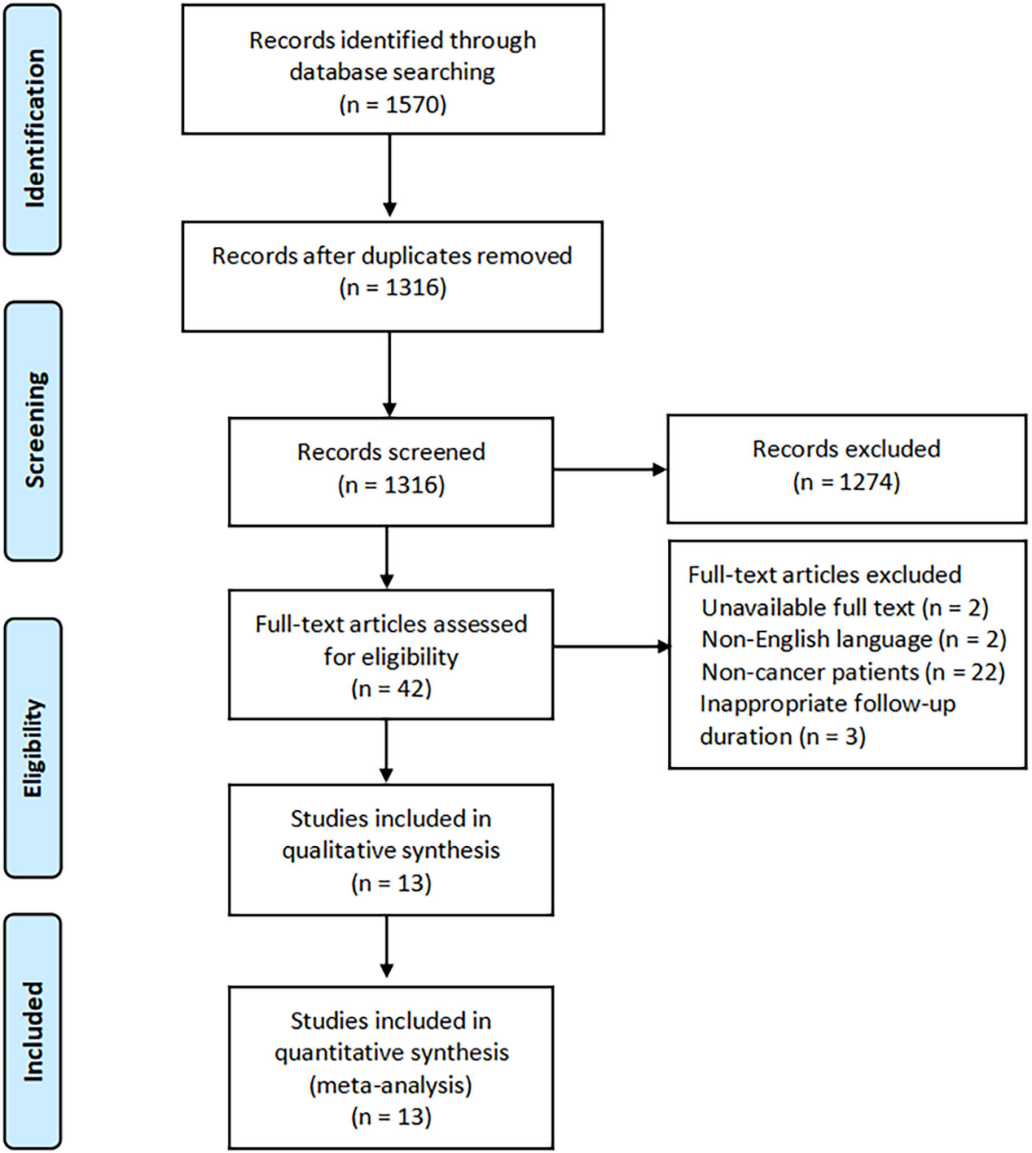
The study selection flowchart.

A total of 1,570 records were identified from the initial database search, and 254 duplicate papers were removed. Of these, 1,316 records remained, and 1,274 records were excluded after screening the titles or abstracts. Furthermore, 42 records were needed to read the full text, and ultimately, a total of 13 studies (17, 24–35), including 6,653 cancer patients, were included in the meta-analysis. The study selection flowchart is shown in **Figure 1**.

### 3.2 Characteristics of the included studies

These studies were conducted in two studies each in the USA (24, 25), the UK (28, 32), France (27, 34), and Spain (33, 35), and one each in China (29), India (26), and Italy (30). Two studies (17, 31) both used cross-sectional data from the OnCoVID study, which is an active European registry study enrolling consecutive patients from Belgium, France, Germany, Italy, Spain, and the UK (17). All the patients were followed up for more than 4 weeks, and the follow-up duration ranged from 28 days to 14 months. Only 10 studies (17, 24, 25, 27–32, 35) used long COVID in cancer patients as an outcome measure; others (26, 33, 34) just reported the prevalence of long COVID in cancer patients. One study (32) included 94 hematology patients, 90 of whom had a hematological malignancy, and the remain 4 had non-malignant hematological diseases. Six studies (28, 30, 32–35) included patients with a diagnosis of COVID-19 and did not explicitly mention how to test for a COVID-19 diagnosis. The characteristics of the included studies are presented in **Table 1**.

**Table 1:** Characteristics of the included studies (*n* = 13).

Study ID	Study design	Country	Follow-up	Test for diagnosis	Sample	Cancer patient infected with SARS-COV-2		
						*N*	Age	Female
Dagher, 2023 (25)	Prospective	USA	Median 7 months	NR	312	312	57 (18,86)	181 (58%)
Martinez-Lopez, 2023 (35)	Cohort, Prospective	Spain	NR	RT-PCR	1,166	278	67 (IQR 54.5–76)	107 (54%)
Lasagna, 2023 (30)	Cohort, Prospective	Italy	NR	Antigen test, PCR	97	97	63 (IQR 16)	58 (60%)
Willan, 2023 (32)	Prospective	UK	NR	Lateral flow test	94	57	NR	NR
Nair, 2023 (26)	Cohort, Prospective	India	Week 6 and week 12 after the patient was confirmed to have a negative COVID-19 test	RT-PCR	414	10	NR	NR
Fankuchen, 2023 (24)	Cohort, Prospective	USA	Median 360 days	NR	359	75	71 (61,83)	34 (45%)
Fernandez, 2022 (33)	Retrospective	Spain	NR	antigen test, serology test, PCR	110,726	3,579	NR	NR
Cortellini, 2022 (31)	Retrospective	Multi center	Median 9.9 months (95% CI, 8.8–11.3)	RT-PCR	186	186	NR	92 (49%)
Hajjaji, 2022 (27)	Cohort, Prospective	France	more than 6 months	NR	2,116	168	NR	120 (71%)
Robineau, 2022 (34)	Cohort, Prospective	France	NR	PCR	3,972	163	NR	NR
Monroy-Iglesias, 2022 (28)	Prospective	UK	NR	RT-PCR	80	80	58 (SD 14)	27 (34%)
Pinato, 2021 (17)	Retrospective	Multi center	28–329 days	NR	1,557	1,557	NR	803 (52%)
Chen, 2021 (29)	Cohort, Prospective	China	Median 12.2 months (IQR, 12.1–12.6)	RT-PCR	664	114	NR	NR

COVID-19, coronavirus disease 2019; SD, standard deviation; IQR, interquartile range; CI, confidence interval; NR, not reported; RT-PCR, reverse transcription−polymerase chain reaction; PCR, polymerase chain reaction.

### 3.3 Methodological quality of the included studies

Nine studies (17, 24, 25, 27–29, 31, 32) with scores above 7 points indicated that most studies were of a high quality. One study (30) scored 6 points, two studies (33, 34) scored 4 points, and one (26) scored 2 points. Four studies (26, 27, 30, 33) did not report the number of patients lost to follow-up, and two retrospective studies (17, 31) were not applicable, which is the main problem for quality assessment. Second, five studies (24, 26, 28, 30, 32) did not have sufficient sample size. Four studies (26, 29, 33, 34) did not report the demographic information of cancer patients in detail. The detailed assessment results are shown in **Supplementary Table S2**.

### 3.4 Meta-analysis

#### 3.4.1 Prevalence of long COVID

**Figure 2:**
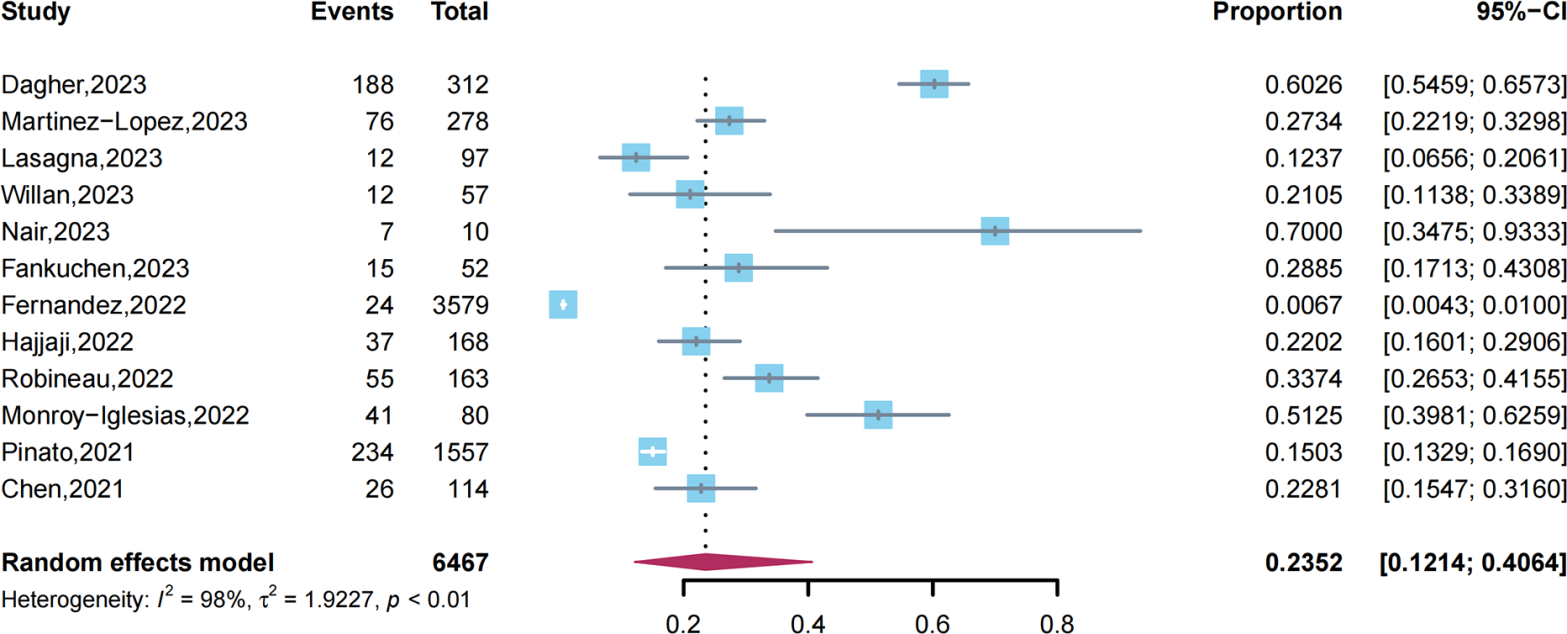
Prevalence of long COVID among cancer patients.

Because two studies (17, 31) used a portion of data from the OnCoVID study, only the study with a larger sample size was included in the meta-analysis to prevent duplication of patients. We performed a pooled analysis of 12 studies comprising 6,467 patients. The results of the random-effects model (*I²* = 98.3%) showed that approximately 23.52% (95% CI, 12.14% to 40.64%) of cancer patients experienced long COVID after acute SARS-CoV-2 infection (**Figure 2**).

#### 3.4.2 Sensitivity analysis and publication bias

**Figure 3:**
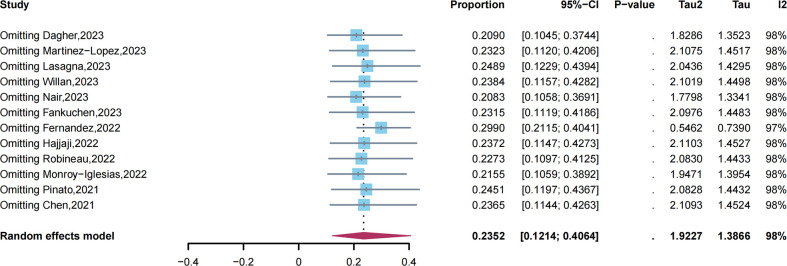
Sensitivity analysis of long COVID prevalence.

We conducted a sensitivity analysis by eliminating one study at a time (**Figure 3**). After the removal of one study (33), heterogeneity was significantly reduced from *I²* = 98.3% to *I² =* 96.7%, and the pooled estimated prevalence of long COVID increased from 23.52% to 29.9%. This suggested that the study may have a potential impact on the results. Egger’s tests were performed for the prevalence of long COVID, and the results revealed that no publication bias was observed (*p* = 0.88).

#### 3.4.3 Subgroup analysis

Five studies (24, 26, 29, 33, 34) reported long COVID in both cancer patients and patients with no cancer diagnosis. The results of the random-effects model (*I²* = 73.3%) revealed that there was no statistical difference between cancer patients and non-cancer patients with SARS-COV-2 infection (OR = 1.24; 95% CI, 0.78 to 1.97; *p* = 0.37).

Four studies (24, 30, 32, 35) reported a long-term follow-up of 3 months. The results of the random-effects model (*I²* = 50.7%) revealed that approximately 20.51% (95% CI, 15.91% to 26.03%) of cancer patients experienced long COVID.

Four studies (24, 27, 31, 35) reported a long-term follow-up of 6 months. The results of the random-effects model (*I²* = 70%) showed that approximately 15.79% (95% CI, 11.39% to 21.47%) of cancer patients experienced long COVID.

Three studies (24, 29, 31) reported a long-term follow-up of 12 months. The proportion did not satisfy a normal distribution (*p* = 0.038). After using Freeman–Tukey double-arcsine transformation, the data conformed to a normal distribution. The results of the random-effects model (*I²* = 79.7%) showed that approximately 12.54% (95% CI, 6.38% to 23.18%) of cancer patients still experienced long COVID. The results of subgroup analysis are shown in the **Supplementary Materials.**

#### 3.4.4 Risk factors for long COVID

Six studies (17, 25, 27, 28, 30, 35) involved five risk factors that were reported by more than three studies. Three studies (27, 28, 35) compared patients with comorbidities to patients without comorbidities. The results of the fixed-effects model (*I²* = 0%) revealed that patients with comorbidities had a significantly higher risk of experiencing long COVID (OR = 1.72; 95% CI, 1.09 to 2.7; *p* = 0.019). In addition, another study (17) indicated that cancer patients with more than two comorbidities were at a higher risk of experiencing long COVID (*p* < 0.001). Five studies (17, 25, 27, 28, 35) reported the risk between hospitalized and non-hospitalized patients with cancer. The results of the random-effects model (*I²* = 88.6%) revealed that no significant difference was observed (OR = 1.84; 95% CI, 0.89 to 3.80; *p* = 0.102). However, a study (32) involving 94 hematological patients indicated that hospitalization would increase the risk of long COVID. However, there were four patients with non-malignant hematological diseases that could not be eliminated. The study was not included in the combined sample. Furthermore, no significant difference was observed in sex, primary tumor, or tumor stage (**Table 2**).

**Table 2:** Risk factors for long COVID in cancer patients.

	No. of studies	Sample size	*I* ^2^, %	OR (95% CI)	*p*-value
Sex (male/female)	6	1,144/1,338	67.1%	0.78 [0.52; 1.17]	0.224
Hospitalization (yes/no)	5	1,405/980	88.6%	1.84 [0.89; 3.80]	0.102
Primary tumor (solid/hematological)	3	1,618/315	0%	1.05 [0.77; 1.42]	0.776
Tumor stage (advanced/local or locoregional)	3	812/815	0%	0.95 [0.73; 1.24]	0.723
Comorbidities (≧1/0)	3	340/155	0%	1.72 [1.09; 2.70]	0.019

OR, odds ratio; Cl, confidence interval.

### 3.5 Systematic review

#### 3.5.1 Risk factors for long COVID

Because of the different criteria, we could not perform a meta-analysis of the associations. Five studies (17, 27, 28, 30, 35) contrasted different age intervals, and only one study indicated that there was a statistically significant difference in that cancer patients over the age of 65 years were more likely to have long COVID (*p* = 0.048) [17]. Different anti-tumor treatment strategies were not statistically associated with long COVID in three studies (17, 27, 35). Two studies revealed that obese patients had a significantly higher risk of experiencing long COVID (27, 30). Pinato et al. (17) reported that patients with long COVID were more likely to have a history of smoking than those with no history (*p* < 0.001), and reported that the distribution of primary tumor was also significantly different (*p* = 0.048). Lasagna et al. (30) reported that type 2 diabetes mellitus (T2DM) is associated with long COVID (*p* = 0.004).

#### 3.5.2 Symptoms with a higher frequency

A total of six studies (17, 25, 27–30) reported the long COVID-specific symptoms with a higher frequency. Fatigue was the most common symptom that occurred in cancer patients after SARS-CoV-2 infection. Respiratory symptoms, myalgia, sleep disturbance, and cognitive impairment were reported in two or more studies. Dyspnea, brain fog, dizziness, loss of taste or smell, weight loss, and gastrointestinal symptoms were also reported in only one study (**Table 3**).

**Table 3:** The top four reported symptoms in the included studies.

Study ID	Top 4 symptoms with the highest frequency	
	The most common symptom	Others
Dagher, 2023 (25)	Fatigue	Sleep disturbance, myalgia, gastrointestinal
Lasagna, 2023 (30)	Fatigue	Brain fog, myalgia, respiratory symptoms
Hajjaji, 2022 (27)	Fatigue	Loss of taste or smell, myalgia, dizziness
Monroy-Iglesias, 2022 (28)	Fatigue	Respiratory symptoms, cognitive impairment, sleep disturbance
Pinato, 2021 (17)	Respiratory symptoms	Fatigue, weight loss, cognitive symptoms
Chen, 2021 (29)	Respiratory symptoms	Dyspnea, myalgia, fatigue

#### 3.5.3 Outcomes after COVID-19

Only three studies (17, 25, 31) presented outcomes after COVID-19. Dagher et al. (25) reported that no long COVID cancer patient had a higher mortality beyond 30 days of initial COVID-19 diagnosis (*p* < 0.001). Pinato et al. (17) reported that long COVID may cause more adjustments or discontinuations of systemic anti-cancer therapy (SACT) (*p* = 0.021) and the discontinuations of SACT were confirmed to be independently associated with an increased risk of death (*p* = 0.002). However, Cortellini et al. (31) reported that disease progression at 6 months had no significant difference between patients who experienced long COVID and those who did not experience long COVID (*p* = 0.139).

## 4 Discussion

This systematic review and meta-analysis investigated the prevalence of long COVID in cancer patients. We found that approximately 23.52% of cancer patients reported experiencing long COVID after acute SARS-CoV-2 infection. A recent meta-analysis of 137 studies revealed that the prevalence of any long COVID was more than 40% in healthy adults (36). However, our finding did not observe any significant differences between cancer patients and those with no cancer.

We also found that long COVID symptoms decreased over time as the follow-up duration increased. This is similar to findings from studies of other populations (37). These findings indicate that long COVID may result in complete recovery even if the time until full recovery is long. Notably, approximately 12.54% of cancer patients reported experiencing long COVID after 12 months of being infected with SARS-CoV-2. In a longitudinal cohort study, COVID-19 survivors had a markedly lower health status than the general population at 2 years (38). Long COVID has been shown to affect multiple organ systems. A long-lasting reduction in vascular density, specifically affecting small capillaries, was found in patients with long COVID compared with controls 18 months after infection, with the potential to affect oxygen delivery (39). Compared with non-COVID-19 controls, people with long COVID have been found to have gut dysbiosis lasting at least 14 months (40).

Owing to the lack of complete data on long COVID symptoms in each study, we used a qualitative descriptive approach to summarize symptoms with a higher frequency. Our findings indicated that fatigue is the symptom with the highest frequency, followed by respiratory symptoms, myalgia, and sleep disturbance. A similar meta-analysis investigated the symptoms of post-COVID-19 patients from 3 to 12 months after COVID-19 hospitalization and revealed that more than 40% of patients experienced fatigue and breathlessness, followed by cough, sleep disturbance, depression, and loss of taste or smell. In addition, long COVID can impact patients’ quality of life (41). A meta-analysis indicated that long COVID patients may experience persistent decreases in quality of life up to 6 months after infection (4). Persistent symptoms and decreased quality of life may not be significantly associated with disease severity (42).

Significant differences in long COVID risk between patients with comorbidities and patients with no comorbidities were found in this meta-analysis. A similar phenomenon was observed in a previous cohort study conducted in China (5) that reported that having two or more comorbidities resulted in a higher risk of developing long COVID at the 6-month follow-up. There were studies that showed that obesity or having T2DM was significantly associated with a higher risk of long COVID, as previous meta-analysis research had suggested (43). Obesity and long COVID share a metabolic proinflammatory state that promotes associated signs and symptoms to linger for a prolonged period of time (44). T2DM has a bidirectional relationship with COVID-19, and COVID-19 itself has been postulated to cause diabetes and to worsen glycemic control in pre-existing diabetes (45). A long duration of uncontrolled diabetes causes organ damage, and in particular, microvascular injury may contribute to long COVID (46). In addition, having a history of smoking may be associated with experiencing long COVID. Smoke has been shown to be a significant risk factor for both long COVID and severe acute COVID-19 (47, 48). Cigarette smoke exposure increases ACE2, which may increase the risk of developing severe COVID-19 as well as a higher mortality (49–51). It is still controversial whether older age is an independent risk factor for long COVID in cancer patients. A meta-analysis revealed that individuals 40 to 69 years old and those 70 years or older are at equally high risk of long COVID (43). There is an assumption that it is difficult for older patients who are at high risk of long COVID to survive from acute COVID-19 infection (52). No association was observed between long COVID and sex, which is inconsistent with the findings of previous studies. Sylvester et al. (53) reported that the likelihood of having long COVID syndrome was significantly greater among female individuals. A UK study including more than 480,000 COVID-19 patients indicated that female sex was a risk factor for long COVID (54). Further research is needed to explore whether female sex is a characteristic of long COVID in cancer patients or it is the result of heterogeneity and bias. In addition, we found that there were no significant differences between the difference in primary tumor, anti-tumor treatment strategies, or tumor stage.

This systematic review and meta-analysis has several strengths. First, this study is the first study to evaluate the association between long COVID and cancer. Second, we also performed subgroup analysis according to the follow-up time and detected publication bias. Third, the only study that included both patients with risk factors and patients without risk factors was included in the analysis to decrease the heterogeneity of the different population. However, certain limitations still exist in our study. First, the included studies and sample size were limited. Although cancer patients should be more focused on and protected during the COVID-19 pandemic, few studies have reported the symptoms of long COVID among cancer patients. Second, there was considerable heterogeneity among the studies, possibly caused by differences in strain, race, geographic location, time of onset of illness, and measurement tools. Third, most studies did not show all of the symptoms they investigated, making it difficult to analyze. The full results of the studies should be encouraged to be indicated. Fourth, few studies have focused on the risk factors for long COVID in cancer patients or the disease outcomes after infection.

## 5 Conclusion

In our findings, nearly a quarter of cancer patients will experience long COVID after surviving from SARS-CoV-2 infection, and this may even last for 1 year or longer. Fatigue, myalgia, cough, and sleep disturbance need to be more addressed and managed to reduce the symptom burden in cancer patients and improve their quality of life. Patients with comorbidities are at a high risk of developing long COVID. However, owing to the above limitations, our results need to be further verified by more studies with higher methodological quality.

## Data Availability

The original contributions presented in the study are included in the article/**Supplementary Material**. Further inquiries can be directed to the corresponding author.
