# Supplementary material for: Prevalence and risk factors for long COVID among cancer patients: a systematic review and meta-analysis
Source: Front Oncol. 2025 Jan 15;14:1506366. doi: 10.3389/fonc.2024.1506366 (PMC11774732; doi:10.3389/fonc.2024.1506366)
Supplement: Supplementary file 1 [file DataSheet1.docx]

**Supplementary file**

**Content**

**Supplementary Table S1. Search strategy**

**Supplementary Table S2. Risk of bias assessments of the included studies**

**Supplementary figure 1. Prevalence of long COVID-19 in cancer patients versus non-cancer patients for 3 months**

**Supplementary figure 2. [Prevalence](https://pubmed.ncbi.nlm.nih.gov/36931142/) of long COVID among cancer patients in 3 months follow-up duration**

**Supplementary figure 3. [Prevalence](https://pubmed.ncbi.nlm.nih.gov/36931142/) of long COVID among cancer patients in 6 months follow-up duration**

**Supplementary figure 4. [Prevalence](https://pubmed.ncbi.nlm.nih.gov/36931142/) of long COVID among cancer patients in 12 months follow-up duration**

**1 Supplementary Figures and Tables**

**1.1 Supplementary Tables**

**Supplementary Table S1. Search strategy**

**PubMed**:

| #1 | (((((((((((((((((((((((((((((((((((COVID-19[MeSH Terms]) OR (COVID 19 Pandemic[Title/Abstract])) OR (COVID-19 Pandemics[Title/Abstract])) OR (Pandemic, COVID-19[Title/Abstract])) OR (COVID-19 Pandemic[Title/Abstract])) OR (COVID-19 Virus Infections[Title/Abstract])) OR (Coronavirus Disease 19[Title/Abstract])) OR (Coronavirus Disease-19[Title/Abstract])) OR (COVID-19 Virus Disease[Title/Abstract])) OR (Infection, COVID-19 Virus[Title/Abstract])) OR (SARS-CoV-2 Infections[Title/Abstract])) OR (Disease 2019, Coronavirus[Title/Abstract])) OR (SARS-CoV-2 Infection[Title/Abstract])) OR (2019 nCoV Disease[Title/Abstract])) OR (Virus Infection, COVID-19[Title/Abstract])) OR (Disease, COVID-19 Virus[Title/Abstract])) OR (COVID19[Title/Abstract])) OR (2019 nCoV Infection[Title/Abstract])) OR (2019-nCoV Infections[Title/Abstract])) OR (COVID-19 Virus Diseases[Title/Abstract])) OR (Disease, 2019-nCoV[Title/Abstract])) OR (SARS CoV 2 Infection[Title/Abstract])) OR (2019-nCoV Disease[Title/Abstract])) OR (Virus Disease, COVID-19[Title/Abstract])) OR (2019-nCoV Diseases[Title/Abstract])) OR (COVID 19 Virus Infection[Title/Abstract])) OR (Infection, 2019-nCoV[Title/Abstract])) OR (2019 Novel Coronavirus Infection[Title/Abstract])) OR (COVID 19[Title/Abstract])) OR (2019 Novel Coronavirus Disease[Title/Abstract])) OR (2019-nCoV Infection[Title/Abstract])) OR (COVID-19 Virus Infection[Title/Abstract])) OR (COVID 19 Virus Disease[Title/Abstract])) OR (Infection, SARS-CoV-2[Title/Abstract])) OR (Severe Acute Respiratory Syndrome Coronavirus 2 Infection[Title/Abstract])) OR (SARS Coronavirus 2 Infection; Coronavirus Disease 2019[Title/Abstract]) | 398610 |
| --- | --- | --- |
| #2 | ((((((((((((((((((Post-Acute COVID-19 Syndrome[MeSH Terms]) OR (Long-Haul COVIDs[Title/Abstract])) OR (Long Haul COVID 19[Title/Abstract])) OR (Post-COVID Condition[Title/Abstract])) OR (Post-Acute Sequelae of SARS-CoV-2 Infection[Title/Abstract])) OR (Long Haul COVID-19s[Title/Abstract])) OR (Post-COVID Conditions[Title/Abstract])) OR (COVID-19 Syndrome, Post-Acute[Title/Abstract])) OR (Long Haul COVID[Title/Abstract])) OR (Post Acute Sequelae of SARS CoV 2 Infection[Title/Abstract])) OR (Post-Acute COVID-19 Syndromes[Title/Abstract])) OR (COVID-19, Long Haul[Title/Abstract])) OR (Long COVID[Title/Abstract])) OR (Post Acute COVID 19 Syndrome[Title/Abstract])) OR (Post COVID Conditions[Title/Abstract])) OR (Long Haul COVID-19[Title/Abstract])) OR (Post Acute COVID-19 Syndrome[Title/Abstract])) OR (Long-Haul COVID[Title/Abstract])) OR (COVID, Long-Haul[Title/Abstract]) OR ("long-term Covid"[Title/Abstract] OR long-term[Title/Abstract] OR consequence[Title/Abstract] OR "long-term impact"[Title/Abstract] OR "long-term effect"[Title/Abstract] OR "post-acute"[Title/Abstract] OR long-tail[Title/Abstract] OR persist[Title/Abstract] OR "chronic-COVID"[Title/Abstract] OR "long-COVID"[Title/Abstract] OR post-discharge[Title/Abstract] OR postdischarge[Title/Abstract] OR "prolonged symptom"[Title/Abstract] OR "long-haul" [Title/Abstract]) | 1277591 |
| #3 | (Signs and Symptoms[MeSH Terms] OR symptom[Title/Abstract] OR "clinical features"[Title/Abstract] OR signs[Title/Abstract] OR characteristic[Title/Abstract] OR sequela[Title/Abstract] OR complication[Title/Abstract]) | 3764403 |
| #4 | ((((((((((((((((((Neoplasms[MeSH Terms]) OR (Neoplasm[Title/Abstract])) OR (Neoplasias[Title/Abstract])) OR (Tumors[Title/Abstract])) OR (Neoplasia[Title/Abstract])) OR (Tumor[Title/Abstract])) OR (Benign Neoplasms[Title/Abstract])) OR (Neoplasm, Benign[Title/Abstract]))) OR (Neoplasms, Benign[Title/Abstract])) OR (Benign Neoplasm[Title/Abstract])) OR (Cancers[Title/Abstract])) OR (Neoplasms, Malignant[Title/Abstract])) OR (Malignant Neoplasms[Title/Abstract])) OR (Neoplasm, Malignant[Title/Abstract])) OR (Malignancy[Title/Abstract])) OR (Malignancies[Title/Abstract])) OR (Malignant Neoplasm[Title/Abstract])) OR (Cancer[Title/Abstract]) | 5043932 |
| #5 | #1 AND #2 AND #3 AND #4 | 138 |

**Web of Science:**

| #1 | (((TS=(COVID-19 OR COVID 19 Pandemic OR COVID-19 Pandemics OR Pandemic, COVID-19 OR COVID-19 Pandemic OR COVID-19 Virus Infections OR Coronavirus Disease 19 OR Coronavirus Disease 19 OR COVID-19 Virus Disease OR COVID-19 Virus Disease OR Infection, COVID-19 Virus OR SARS-CoV-2 Infections OR Disease 2019, Coronavirus OR SARS-CoV-2 Infection OR 2019 nCoV Disease OR Virus Infection, COVID-19 OR Disease, COVID-19 Virus OR COVID19 OR 2019 nCoV Infection OR 2019 nCoV Infection OR 2019-nCoV Infections OR COVID-19 Virus Diseases OR Disease, 2019-nCoV OR SARS CoV 2 Infection OR 2019-nCoV Disease OR Virus Disease, COVID-19 OR 2019-nCoV Diseases OR Virus Disease, COVID-19 OR 2019-nCoV Diseases OR COVID 19 Virus Infection OR Infection, 2019-nCoV OR 2019 Novel Coronavirus Infection OR COVID 19 OR 2019 Novel Coronavirus Disease OR 2019-nCoV Infection OR COVID-19 Virus Infection OR COVID 19 Virus Disease OR Infection, SARS-CoV-2 OR Severe Acute Respiratory Syndrome Coronavirus 2 Infection OR SARS Coronavirus 2 Infection OR coronal)) AND TS=(Neoplasms OR Neoplasm OR Neoplasias OR Tumors OR Neoplasia OR Tumor OR Cancers OR Neoplasms, Malignant OR Malignant Neoplasms OR Neoplasm, Malignant OR Malignant Neoplasms OR Neoplasm, Malignant OR Malignancy OR Malignancies OR Malignant Neoplasm OR Cancer)) AND TS=(sign OR signs OR symptoms OR symptom OR "clinical features" OR characteristic OR sequela OR complication)) AND TS=(Post-Acute COVID-19 Syndrome OR Long-Haul corvids OR Long Haul COVID 19 OR Post-COVID Condition OR Post-Acute Sequelae of SARS-CoV-2 Infection OR Long Haul COVID-19s OR Post-COVID Conditions OR COVID-19 Syndrome, Post-Acute OR Long Haul COVID OR Post Acute Sequelae of SARS CoV 2 Infection OR Post-Acute COVID-19 Syndromes OR COVID-19, Long Haul OR Long COVID OR Post Acute COVID 19 Syndrome OR Post COVID Conditions OR Long Haul COVID-19 OR Post Acute COVID-19 Syndrome OR Long-Haul COVID OR COVID, Long-Haul OR "long-term Covid" OR long-term OR consequence OR "long-term impact" OR "long-term effect" OR "post-acute" OR long-tail OR persist OR "chronic-COVID" OR "long-COVID" OR post-discharge OR postdischarge OR "prolonged symptom" OR "long-haul") | 1032 |
| --- | --- | --- |

**Embase:**

| #1 | (neoplasms:ab,ti OR neoplasm:ab,ti OR neoplasias:ab,ti OR tumors:ab,ti OR neoplasia:ab,ti OR tumor:ab,ti OR cancers:ab,ti OR 'neoplasms, malignant':ab,ti OR 'malignant neoplasms':ab,ti OR 'neoplasm, malignant':ab,ti OR malignancy:ab,ti OR malignancies:ab,ti OR 'malignant neoplasm':ab,ti OR cancer:ab,ti) AND ('covid 19 pandemic':ab,ti OR 'covid-19 pandemics':ab,ti OR 'pandemic, covid-19':ab,ti OR 'covid-19 pandemic':ab,ti OR 'covid-19 virus infections':ab,ti OR 'coronavirus disease 19':ab,ti OR 'covid-19 virus disease':ab,ti OR 'infection, covid-19 virus':ab,ti OR 'sars-cov-2 infections':ab,ti OR 'disease 2019, coronavirus':ab,ti OR 'sars-cov-2 infection':ab,ti OR '2019 ncov disease':ab,ti OR 'virus infection, covid-19':ab,ti OR 'disease, covid-19 virus':ab,ti OR covid19:ab,ti OR '2019 ncov infection':ab,ti OR '2019-ncov infections':ab,ti OR 'covid-19 virus diseases':ab,ti OR 'disease, 2019-ncov':ab,ti OR 'sars cov 2 infection':ab,ti OR '2019-ncov disease':ab,ti OR 'virus disease, covid-19':ab,ti OR '2019-ncov diseases':ab,ti OR 'covid 19 virus infection':ab,ti OR 'infection, 2019-ncov':ab,ti OR '2019 novel coronavirus infection':ab,ti OR 'covid 19':ab,ti OR '2019 novel coronavirus disease':ab,ti OR '2019-ncov infection':ab,ti OR 'covid-19 virus infection':ab,ti OR 'covid 19 virus disease':ab,ti OR 'infection, sars-cov-2':ab,ti OR 'severe acute respiratory syndrome coronavirus 2 infection':ab,ti OR 'sars coronavirus 2 infection':ab,ti OR coronal:ab,ti) AND (sign:ab,ti OR signs:ab,ti OR symptoms:ab,ti OR symptom:ab,ti OR 'clinical features':ab,ti OR characteristic:ab,ti OR sequela:ab,ti OR complication:ab,ti) AND ('post-acute covid-19 syndrome':ab,ti OR 'long-haul corvids':ab,ti OR 'long haul covid 19':ab,ti OR 'post-covid condition':ab,ti OR 'post-acute sequelae of sars-cov-2 infection':ab,ti OR 'long haul covid-19s':ab,ti OR 'post-covid conditions':ab,ti OR 'covid-19 syndrome, post-acute':ab,ti OR 'long haul covid':ab,ti OR 'post acute sequelae of sars cov 2 infection':ab,ti OR 'post-acute covid-19 syndromes':ab,ti OR 'covid-19, long haul':ab,ti OR 'long covid':ab,ti OR 'post acute covid 19 syndrome':ab,ti OR 'post covid conditions':ab,ti OR 'long haul covid-19':ab,ti OR 'post acute covid-19 syndrome':ab,ti OR 'long-haul covid':ab,ti OR 'covid, long-haul':ab,ti OR 'long-term covid':ab,ti OR 'long term':ab,ti OR consequence:ab,ti OR 'long-term impact':ab,ti OR 'long-term effect':ab,ti OR 'post-acute':ab,ti OR 'long tail':ab,ti OR persist:ab,ti OR 'chronic-covid':ab,ti OR 'long-covid':ab,ti OR 'post discharge':ab,ti OR postdischarge:ab,ti OR 'prolonged symptom':ab,ti OR 'long-haul':ab,ti) | 400 |
| --- | --- | --- |

**Supplementary Table S2. Risk of bias assessments of the included studies**

| **Study ID** | **Was the sample frame appropriate to address the target population?** | **Were study participants recruited in an appropriate way?** | **Was the sample size adequate?** | **Were the study subjects and setting described in detail?** | **Was data analysis conducted with sufficient coverage of the identified sample?** | **Were valid methods used for the identification of the condition?** | **Was the condition measured in a standard, reliable way for all participants?** | **Was there appropriate statistical analysis?** | **Was the response rate adequate, and if not, was the low response rate managed**  **appropriately?** | **Overall score** |
| --- | --- | --- | --- | --- | --- | --- | --- | --- | --- | --- |
| Dagher,2023 | Y | Y | Y | Y | Y | N | Y | Y | Y | 8 |
| Martinez-Lopez,2023 | Y | Y | Y | Y | Y | Y | Y | Y | Y | 9 |
| Lasagna,2023 | Y | Y | N | Y | Y | Y | N | Y | N | 6 |
| Willan,2023 | Y | Y | N | Y | Y | Y | Y | Y | Y | 8 |
| Nair,2023 | N | N | N | N | N | Y | Y | N | N | 2 |
| Fankuchen,2023 | Y | Y | N | Y | Y | N | Y | Y | Y | 7 |
| Fernandez,2022 | N | Y | Y | N | N | Y | Y | N | N | 4 |
| Cortellini,2022 | Y | Y | Y | Y | Y | Y | Y | Y | NA | 8 |
| Hajjaji,2022 | Y | Y | Y | Y | Y | N | Y | Y | N | 7 |
| Robineau,2022 | N | N | Y | N | N | Y | Y | N | Y | 4 |
| Monroy-Iglesias,2022 | Y | Y | N | Y | Y | Y | Y | Y | Y | 8 |
| Pinato,2021 | Y | Y | Y | Y | Y | Y | Y | Y | NA | 8 |
| Chen,2021 | Y | Y | Y | N | Y | Y | Y | Y | Y | 8 |

Y: yes; N: no; U: unclear; NA: not applicable

**1.2 Supplementary Figures**

**
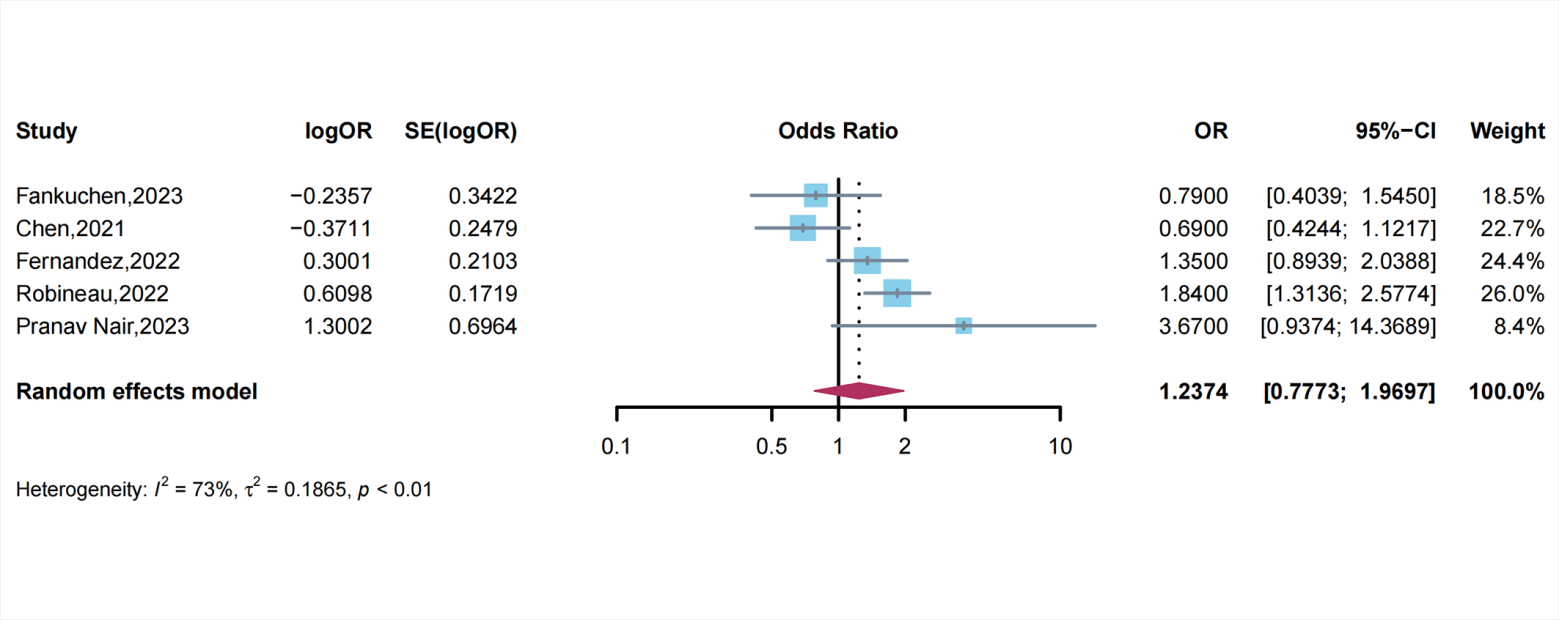
**

**Supplementary figure 1. Prevalence of long COVID-19 in cancer patients versus non-cancer patients**

**
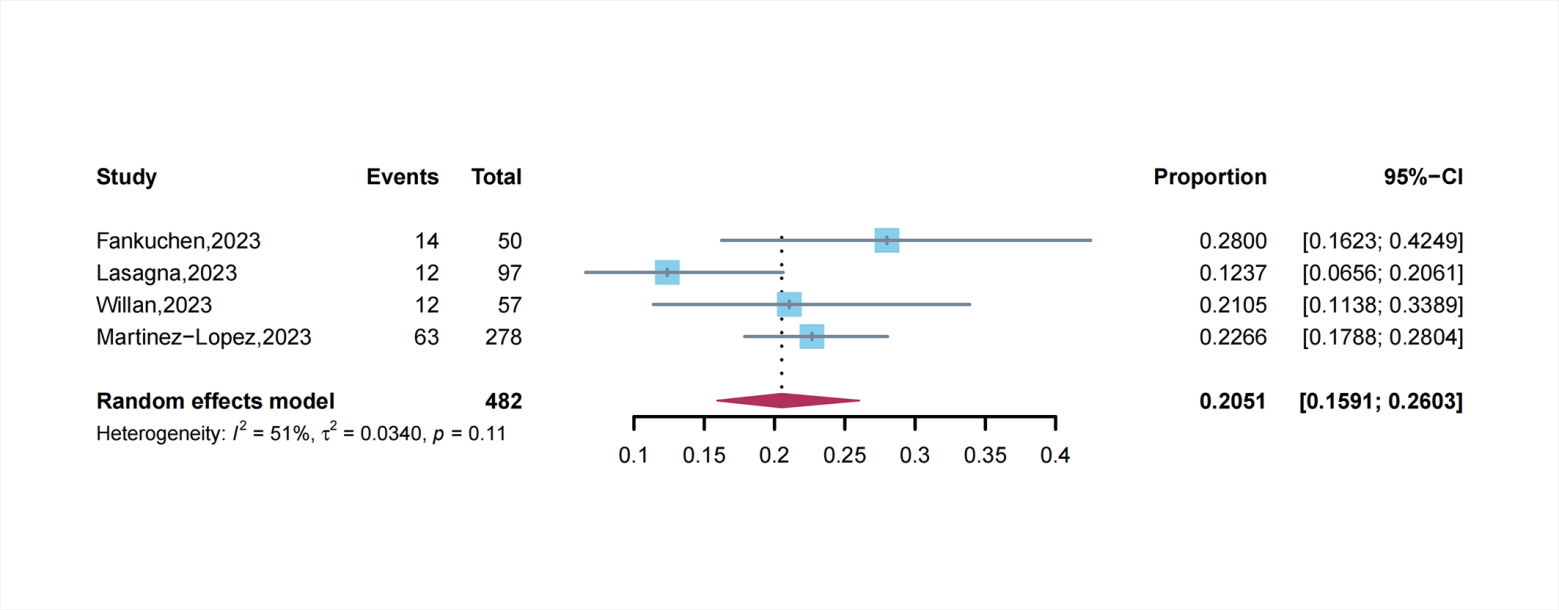
**

**Supplementary figure 2. [Prevalence](https://pubmed.ncbi.nlm.nih.gov/36931142/) of long COVID among cancer patients in 3 months follow-up duration**

**
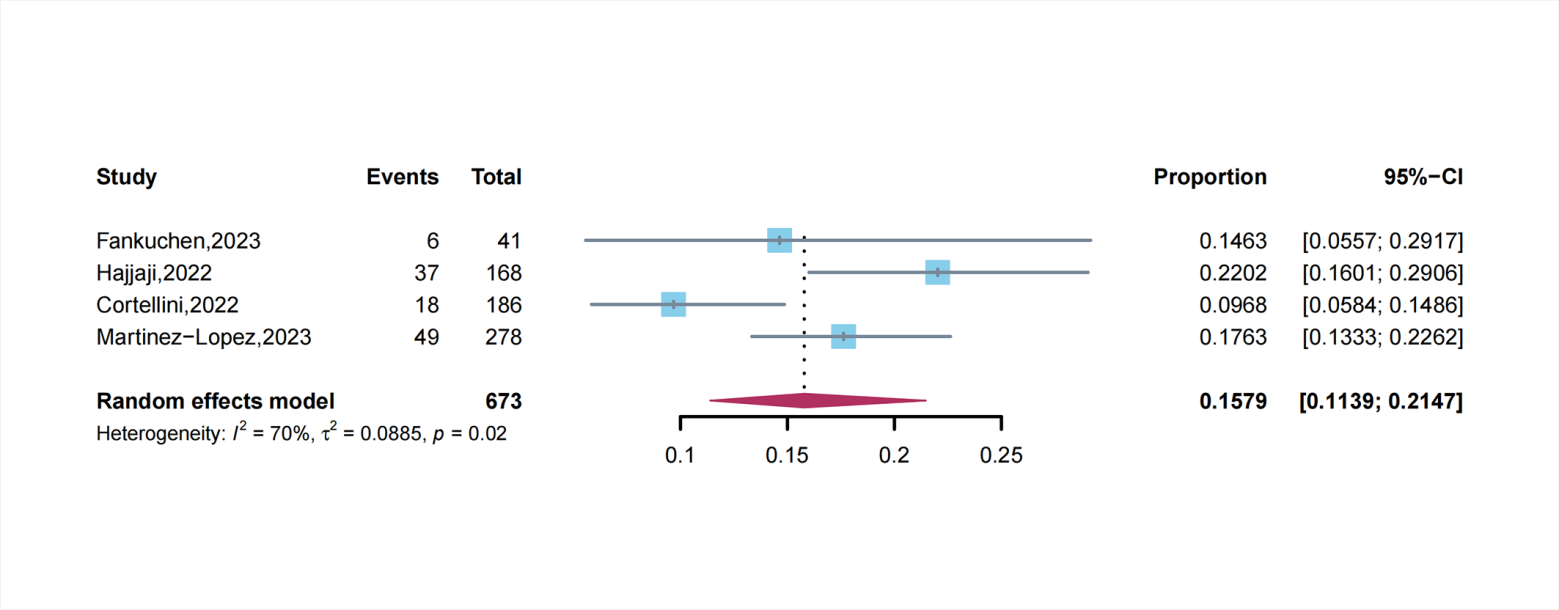
**

**Supplementary figure 3. [Prevalence](https://pubmed.ncbi.nlm.nih.gov/36931142/) of long COVID among cancer patients in 6 months follow-up duration**

**
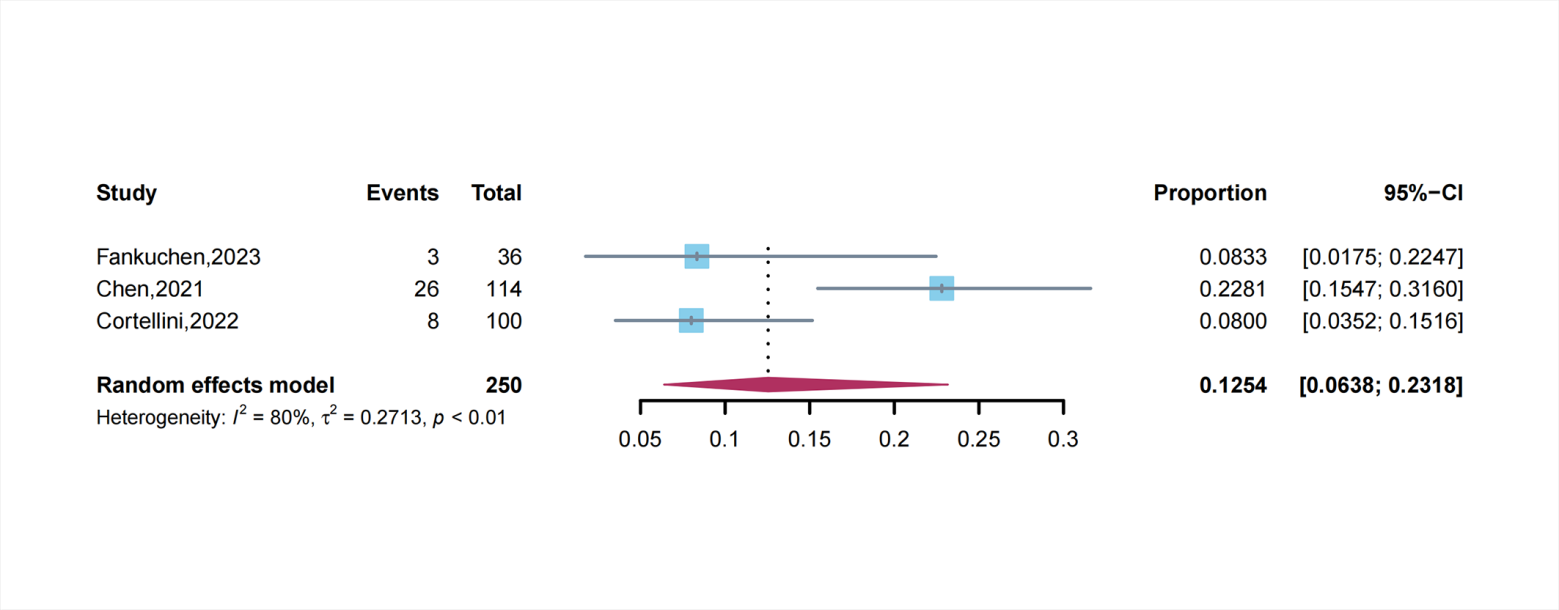
**

**Supplementary figure 4. [Prevalence](https://pubmed.ncbi.nlm.nih.gov/36931142/) of long COVID among cancer patients in 12 months follow-up duration**
